# Neuro-Emotional Technique and Memory Reconsolidation: A Narrative Review and Conceptual Framework for Somatic-Emotional Updating

**DOI:** 10.7759/cureus.111944

**Published:** 2026-07-02

**Authors:** Ryan R Day

**Affiliations:** 1 Integrative Medicine and Mind-Body Research, Ryan Day Chiropractic, Sydney, AUS

**Keywords:** adaptive physiology, allostatic load, interoception, memory reconsolidation, mind-body intervention, neuro-emotional technique, predictive processing, somatic-emotional updating

## Abstract

Neuro-emotional technique (NET) is a structured mind-body intervention used by certified practitioners to identify and address stress-related emotional patterns associated with physiological and behavioural responses. Published NET research has described changes in psychological symptoms, pain, quality of life, brain physiology, inflammatory markers, and blood chemistry, yet the mechanisms underlying these effects remain incompletely defined. This narrative review and conceptual synthesis propose that memory reconsolidation theory and predictive processing provide complementary frameworks for understanding how NET may influence stress-linked somatic-emotional patterns.

Reconsolidation research suggests that previously consolidated emotional memories may become temporarily labile following reactivation if a sufficient prediction error occurs, permitting updating before restabilisation. Predictive processing models propose that emotional learning may be expressed as embodied predictions about threat, safety, self, action, and physiological demand, rather than solely as conscious narrative recall. Taken together, these frameworks suggest that the clinically relevant target of NET may be a body-brain prediction pattern rather than a discrete memory or a fixed physiological state.

This article introduces somatic-emotional updating to describe the proposed modification of emotionally encoded physiological prediction patterns through concurrent emotional activation and contradictory embodied experience. Within this model, NET may create clinical conditions in which a stress-linked somatic-emotional pattern is reactivated while the patient simultaneously experiences a sense of present-time safety, interoceptive attention, practitioner support, and non-threatening somatosensory input. This combination is proposed to constitute embodied mismatch, the clinical expression of prediction error, which may provide conditions consistent with reconsolidation-relevant updating.

The model does not propose that NET has been proven to induce memory reconsolidation. Rather, it identifies structural parallels between the NET clinical sequence and reconsolidation phases and proposes somatic-emotional updating as a theoretically coherent and falsifiable hypothesis for future research. Proposed research priorities include measuring durable changes in target emotional reactivity, autonomic regulation, biological stress markers, and adaptive function following NET, as well as comparing full NET with modified protocols that isolate key procedural elements.

## Introduction and background

Emotional stress is increasingly understood as a biologically active process that can influence autonomic regulation, neuroendocrine activity, immune signalling, pain sensitivity, sleep, behaviour, and recovery capacity. These responses are adaptive when brief and proportionate but may become maladaptive when repeatedly activated or expressed outside the original context. Allostatic load theory provides one framework for understanding how repeated activation of stress mediators may contribute to physiological strain over time [1].

Neuro-emotional technique (NET) is a structured mind-body intervention developed to identify and address stress-related emotional patterns associated with physiological or behavioural responses. Published NET studies and case reports have described changes in psychological symptoms, pain, quality of life, brain physiology, inflammatory markers, blood chemistry, and other biobehavioural indicators [2-4]. However, the mechanisms by which NET may influence emotional and physiological regulation remain incompletely defined.

In clinical practice, NET typically begins with a current concern, symptom, stress response, or body area relevant to the patient’s presentation. The practitioner then uses structured questioning and psychophysiological cueing to identify an associated emotional reality before guiding the patient to maintain awareness of the relevant feeling, phrase, memory, image, or bodily sensation while somatic or spinal input is applied. This clinical sequence suggests that NET may involve emotional activation, interoceptive attention, contextual safety, somatosensory input, and reassessment. These features provide a rationale for a more explicit mechanistic synthesis.

This article proposes that memory reconsolidation and predictive processing provide complementary frameworks for understanding NET. Reconsolidation research suggests that previously consolidated memories can, under specific conditions, become labile after reactivation and open to modification before restabilising. Predictive processing suggests that emotional learning may be expressed as embodied predictions about threat, safety, self, action, and physiological demand [5-9].

The central hypothesis is that NET may facilitate somatic-emotional updating, defined here as the proposed modification of emotionally encoded physiological prediction patterns through concurrent emotional activation and contradictory embodied experience. This article develops that hypothesis by reviewing NET’s clinical structure, mapping it to reconsolidation and predictive processing frameworks, and considering its implications for adaptive physiology and future research.

## Review

Methods/literature selection

This article was developed as a narrative review and hypothesis-generating conceptual synthesis. It was not designed as a systematic review, meta-analysis, or formal efficacy review. The purpose was to integrate literature relevant to NET, memory reconsolidation, predictive processing, interoceptive regulation, manual therapy neurophysiology, and stress-related adaptive physiology in order to generate a testable conceptual model.

Literature searches were conducted during manuscript preparation in May 2026. Sources were identified through targeted searches of PubMed, Google Scholar, and reference lists of relevant articles. Search terms included combinations of ‘Neuro Emotional Technique’, ‘NET’, ‘memory reconsolidation’, ‘prediction error’, ‘predictive processing’, ‘interoception’, ‘somatic marker’, ‘psychoneuroimmunology’, ‘psycho-immune-neuroendocrine’, ‘allostatic load’, ‘manual therapy neurophysiology’, ‘spinal manipulation’, ‘emotional memory’, ‘therapeutic change’, and ‘inhibitory learning’.

Sources were prioritised for inclusion if they addressed NET clinical structure, published NET findings relevant to stress physiology or neurobiology, memory reconsolidation and prediction error, predictive processing and interoceptive inference, allostasis and psychoneuroimmunology, manual therapy neurophysiology, or alternative explanations for clinical change.

Because this was a targeted narrative synthesis rather than a systematic review, sources were not screened using a formal PRISMA workflow, and a prospective numerical screening log was not maintained. For this reason, the number of records screened is not reported. The final reference list includes 32 sources selected for conceptual relevance, methodological importance, or direct relevance to the proposed model. No formal risk-of-bias assessment was performed, and no pooled statistical synthesis was conducted.

Existing NET studies were cited selectively to establish clinical structure, identify findings relevant to the proposed model, and illustrate candidate psychological, neurophysiological, biological, and functional outcome domains. These studies are interpreted as preliminary and mechanistically suggestive rather than as proof that NET induces memory reconsolidation. This manuscript does not aim to catalogue all published NET studies or extract quantitative outcome data across the full evidence base. A comprehensive mapping of NET studies by design, population, intervention characteristics, outcome measures, and reported quantitative findings would require a separate scoping review or systematic review with predefined eligibility criteria, structured data extraction, and formal risk-of-bias assessment.

Overview of NET

NET is a structured mind-body intervention used by certified practitioners to identify and address stress-related emotional patterns associated with physiological or behavioural responses. Published descriptions characterise NET as a 15-step clinical protocol integrating concepts from behavioural psychology, traditional Chinese medicine, psychophysiology, manual muscle testing, and somatic or spinal stimulation [4,10-12].

A central construct in NET is the neuro-emotional complex (NEC), which refers to an unresolved stress-related pattern associated with a patient’s conditioned emotional reality and bodily response. In NET theory, an NEC may arise when an emotionally significant experience becomes linked with a physiological stress response that persists beyond the original context. The clinically relevant target is therefore not only the recalled event itself, but the current emotional and physiological response that becomes active when the associated theme, phrase, memory, or bodily cue is contacted.

In broad clinical terms, NET begins by identifying a current concern, symptom, stress response, or body area relevant to the patient’s presentation. The practitioner then uses structured questioning and manual muscle testing to explore whether a specific emotional reality is associated with the presenting concern. Manual muscle testing is addressed more specifically below as a clinician-guided psychophysiological cueing process rather than a diagnostic test.

Once an emotional reality has been identified, the practitioner guides the patient to hold awareness of the relevant feeling, image, phrase, memory, or bodily sensation. The protocol may then explore contextual features such as the origin, timing, or personal meaning of the emotional pattern. A further feature of NET is the use of somatic or spinal stimulation while the patient maintains awareness of the identified emotional reality. In clinical practice, this may include brief stimulation of spinal regions through gentle manual tapping, instrument-assisted input, or acupoint-related stimulation, depending on practitioner training and scope of practice [11].

The protocol concludes with reassessment. The practitioner rechecks the relevant response, emotional charge, or physiological cue to determine whether the previously identified pattern remains active. Clinical reassessment may include manual muscle testing; subjective report; emotional charge; changes in breathing, posture, or affect; or other observable features of autonomic state. These observations do not establish a mechanism, but they form part of the procedural structure through which NET identifies, applies, and reassesses a targeted intervention.

For this study, the relevant features are emotional activation, interoceptive attention, contextual safety, psychophysiological cueing, somatosensory input, and reassessment. Because this sequence involves activation of a specific emotional pattern followed by somatic intervention and reassessment, memory reconsolidation provides a relevant framework for examining how such patterns might change.

Manual muscle testing as psychophysiological cueing

Manual muscle testing is one of the more debated components of NET. In conventional clinical contexts, manual muscle testing may be used to assess aspects of strength or neuromuscular function. However, scientific concerns are greater when muscle testing is extended beyond musculoskeletal assessment to infer non-musculoskeletal states, hidden emotional content, or specific biomedical diagnoses. Reliability also depends on testing procedure, examiner training, timing, force application, patient response, and the clinical claim being made [13-15].

For this reason, this study does not treat manual muscle testing as a diagnostic test, a measure of historical truth, or a detector of concealed emotional material. Within the proposed model, manual muscle testing is interpreted more conservatively as a clinician-guided psychophysiological cueing process. It may help direct attention toward stimuli, phrases, memories, body areas, or emotional themes that appear relevant within the structured NET sequence, but it does not independently establish mechanism or causality.

This distinction is important for the somatic-emotional updating hypothesis. The proposed mechanism does not depend on manual muscle testing having independent diagnostic validity. Rather, the relevant procedural features are emotional activation, interoceptive attention, contextual safety, somatosensory input, and reassessment. Future component studies should examine whether outcomes differ when manual muscle testing is retained, standardised, modified, or omitted.

Memory reconsolidation theory

Memory consolidation refers to the stabilisation of a newly formed memory after initial encoding. Long-term memories were traditionally understood to remain relatively stable once consolidated. Reconsolidation research challenged this view by showing that previously consolidated memories may become temporarily labile following retrieval and may require restabilisation after reactivation. The landmark study by Nader et al. demonstrated that reactivated fear memories in rats required renewed protein synthesis in the amygdala to persist after retrieval. When protein synthesis was disrupted shortly after reactivation, the previously consolidated fear response was no longer expressed [5].

Subsequent research refined this model and showed that reconsolidation is not automatically triggered by every act of recall. Memory reactivation alone may be insufficient. A key condition appears to be prediction error, meaning a mismatch between what the existing memory predicts and what is actually experienced. Prediction error may signal that the current memory model is incomplete or inaccurate, permitting destabilisation and updating [6,16].

In emotional learning, prediction error may occur when a previously threatening, shame-linked, grief-linked, or helplessness-linked memory is reactivated while the expected emotional or physiological outcome does not occur. The organism encounters the old emotional prediction in a new experiential context. If the mismatch is sufficient, the memory may enter a labile state and become open to modification before reconsolidating [7].

This distinction is clinically important. Extinction learning generally describes the development of a new inhibitory memory that competes with the original emotional learning. Reconsolidation updating, by contrast, refers to the potential modification of the original emotional memory or its associated response.

In this study, reconsolidation theory is used as a hypothesis-generating framework rather than as evidence that NET has been shown to induce reconsolidation. It provides a testable model by specifying what should be observed if NET engages reconsolidation-relevant processes: emotional reactivation should be necessary, embodied mismatch should predict outcome strength, and changes should persist across subjective and physiological measures. Reconsolidation, therefore, helps explain how a pattern may become modifiable, while predictive processing helps clarify what may be modified.

Predictive processing and embodied physiology

Predictive processing models propose that the brain does not passively receive sensory information from the body and environment. Rather, the nervous system continually generates predictions about the likely causes of sensory input and updates those predictions when incoming information differs from expectation. Perception, emotion, action, and bodily regulation may therefore be understood as active inferential processes rather than simple responses to external stimuli.

This model is especially relevant to interoception, the sensing and interpretation of internal bodily signals. Interoceptive models suggest that feelings arising from the body may not reflect raw physiological input alone, but the brain’s prediction of bodily state, constrained by incoming visceral and somatic information. Barrett and Simmons proposed the Embodied Predictive Interoception Coding model, while Seth and Friston describe emotion and selfhood through active interoceptive inference [8,9].

A central feature of predictive processing is prediction error, which refers to the mismatch between expected and actual sensory input. In predictive coding models, higher-level predictions are compared with incoming sensory information, and the resulting error signal may be used to update the model when the current input is not adequately explained. Prediction error is therefore not simply a mistake, but a biologically useful signal that the organism’s current model may require revision [17].

This logic is especially relevant to the somatic-emotional updating model because emotional learning may be expressed through body-brain predictions about threat, safety, self, action, and physiological demand. If prior experience has shaped predictions about threat, safety, self, action, or physiological demand, the body may prepare for expected conditions before conscious appraisal is complete. This anticipatory preparation is consistent with allostatic models of predictive regulation, in which physiological systems adjust in advance of anticipated need rather than merely reacting after disturbance occurs [18]. From this perspective, emotional and somatic responses may reflect the interaction between prior prediction, current interoceptive and sensory input, and the degree of mismatch between them [8,9,17].

**Figure 1:**
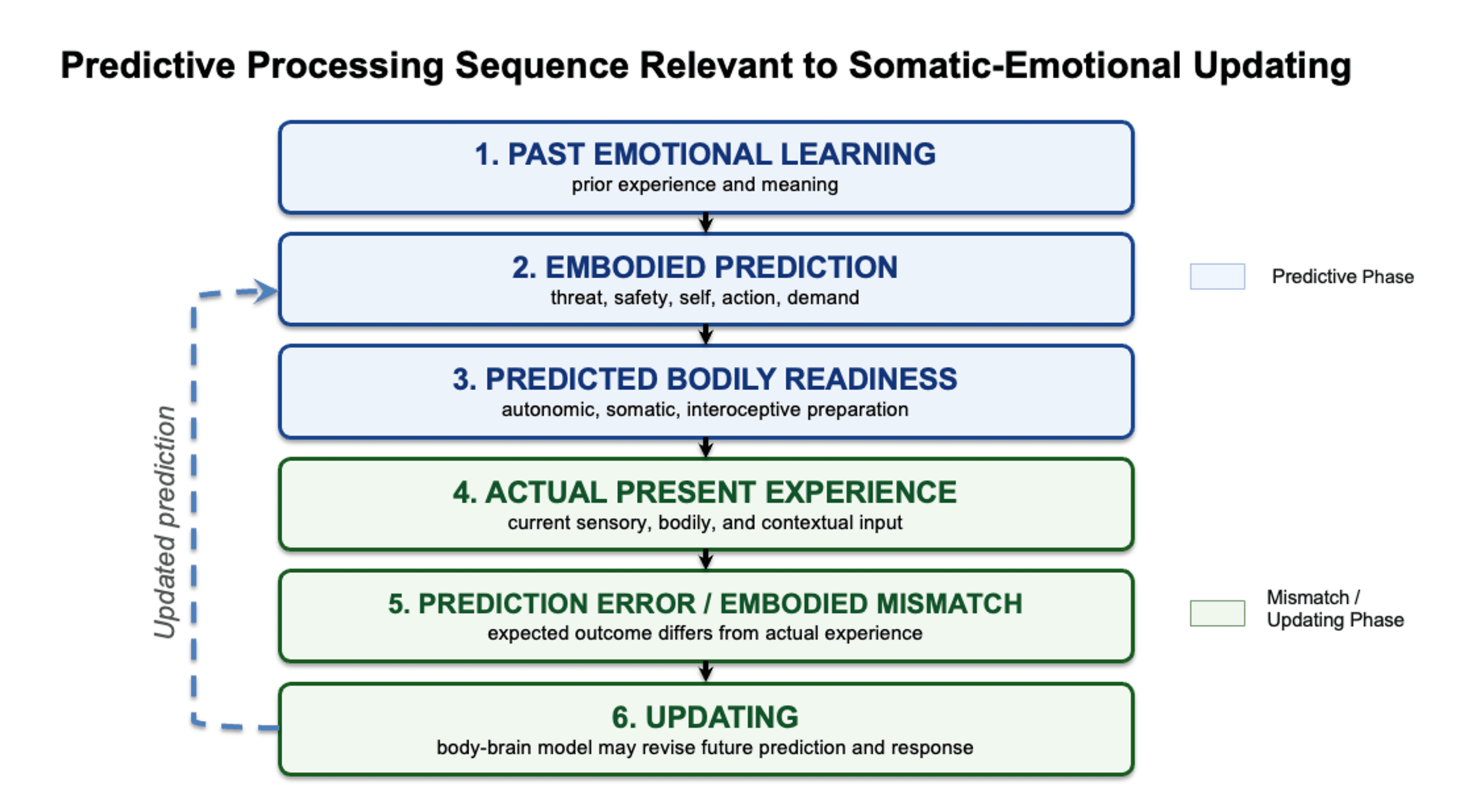
Predictive processing sequence relevant to somatic-emotional updating This figure illustrates a simplified predictive processing sequence relevant to somatic-emotional updating. Prior emotional learning may shape embodied predictions about threat, safety, self, action, and physiological demand. These predictions may influence anticipatory bodily readiness. When predicted bodily readiness is brought into contact with actual present sensory, bodily, and contextual input, prediction error or embodied mismatch may occur if the expected emotional or physiological outcome differs from the actual experience. If the mismatch is sufficient and contextually meaningful, updating may revise future prediction and response, as represented by the feedback loop to embodied prediction [8,9,17,18]. The figure is conceptual and should not be interpreted as evidence that NET induces prediction error, memory reconsolidation, or physiological updating. Credit: This figure was created manually by the author using Microsoft PowerPoint (Microsoft Corp., Redmond, WA) shapes and text only.

For readers unfamiliar with predictive processing, Figure 1 summarises this simplified sequence as it relates to somatic-emotional updating.

In this framework, emotional memory is not confined to explicit autobiographical recall. It may also be expressed as a conditioned pattern of bodily readiness. A cue may activate an embodied prediction before conscious appraisal occurs. Damasio’s somatic marker hypothesis is relevant because it proposes that body-based emotional signals can influence perception, decision-making, and response selection [19]. Allostasis further strengthens this model by describing regulation through anticipation. Chronic stress becomes clinically important because the organism may repeatedly prepare for predicted demands, even when those demands are no longer present or proportionate [18].

For NET, this framework suggests that the clinically relevant target may not be the past event itself, but the present-day prediction pattern associated with that event or emotional reality. Reconsolidation may describe how an emotionally encoded prediction becomes labile and modifiable. Predictive processing describes what may be modified: a body-brain prediction about threat, safety, self, action, or physiological demand. Taken together, reconsolidation and predictive processing suggest that NET may be understood not simply as emotional recall but as a process in which embodied emotional predictions are reactivated and potentially updated.

NET as a somatic-emotional updating process

The preceding sections suggest that emotional memories may be understood not only as conscious narratives but also as embodied prediction patterns. These patterns may include autonomic preparation, interoceptive interpretation, muscle tension, pain sensitivity, respiratory change, behavioural readiness, and immune or endocrine signalling. From this perspective, a clinically relevant emotional memory may include the present physiological prediction that becomes active when the emotional meaning of a prior experience is contacted [8,9].

NET may be conceptualised as a structured clinical process that intentionally brings such patterns into awareness. In NET, the practitioner identifies a current concern, explores a related emotional reality, and directs the patient’s attention toward the associated affective and somatic response. Published descriptions of NET characterise it as an intervention in which unresolved stress patterns or NECs may influence personalised bodily responses connected to a conditioned emotional reality [3].

This study proposes the term somatic-emotional updating to describe a possible mechanism through which NET may exert some of its clinical effects. Somatic-emotional updating refers to the proposed modification of emotionally encoded physiological prediction patterns through concurrent emotional activation and contradictory embodied experience. The term is proposed here as a conceptual construct rather than an established term in the literature.

The construct distinguishes NET from explanations based primarily on catharsis, relaxation, cognitive insight, or symptom suppression. The proposed target of change is not simply the emotional content of a remembered event but the physiological prediction attached to that emotional meaning. A patient may intellectually recognise that a past event is over, yet still experience a bodily response as though the emotional meaning remains current. In reconsolidation terms, durable change may require that the old emotional learning is reactivated and paired with an experience that contradicts the expected outcome [7].

In NET, this contradictory experience may occur through several simultaneous channels. The patient brings attention to an emotionally salient memory, phrase, image, or theme while remaining in a present-time clinical environment. The emotional reality is contacted without the original danger occurring. The patient is supported by the practitioner. The body receives non-threatening somatosensory input. Together, these elements may create an embodied contradiction to the prior emotional prediction. In this study, the term 'embodied mismatch' is used to describe the proposed clinical expression of prediction error during NET, in which the patient contacts an emotionally salient pattern while experiencing a new bodily and relational context.

**Figure 2:**
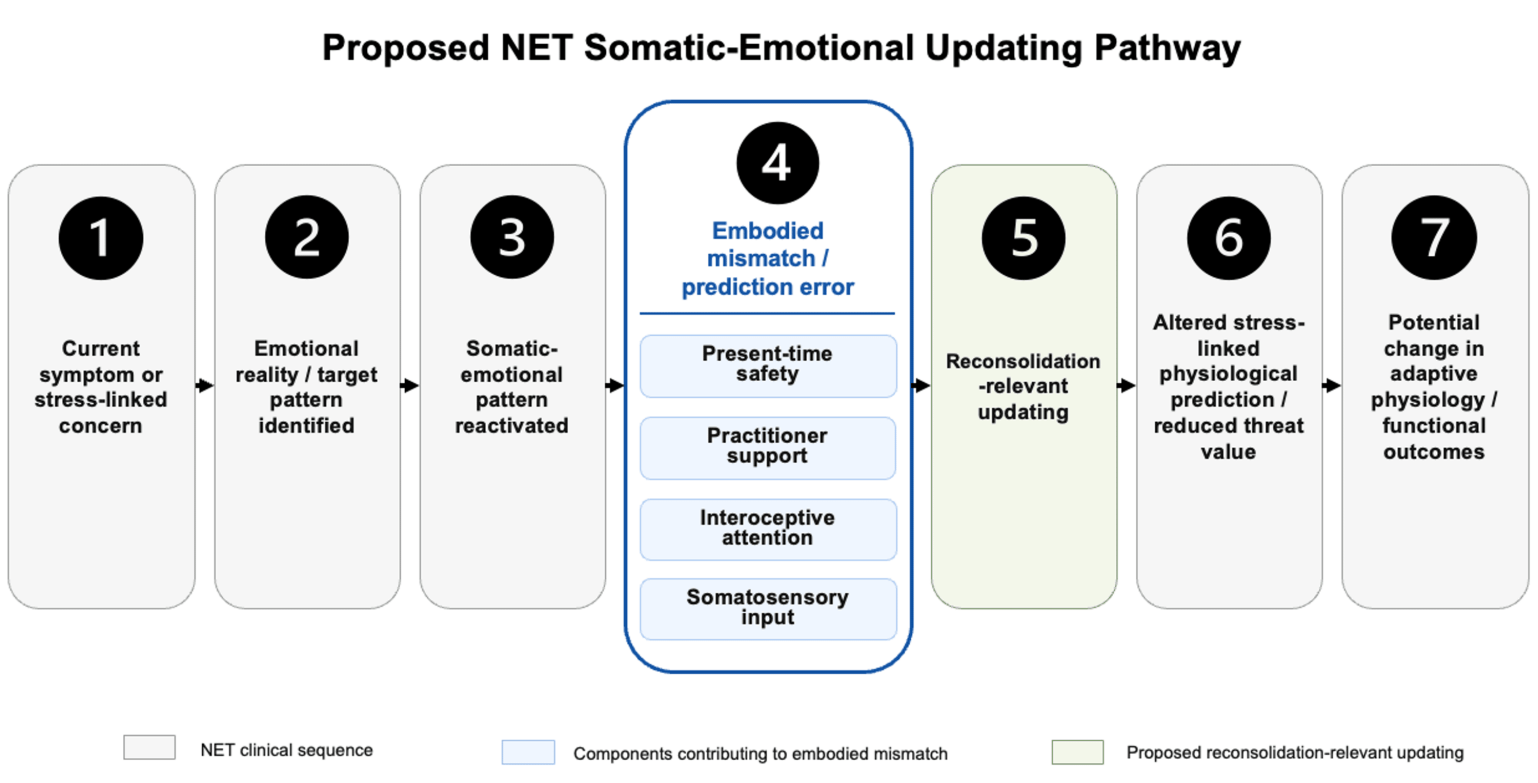
Proposed NET somatic-emotional updating pathway Figure 2 illustrates the proposed pathway by which core neuro-emotional technique (NET) components may create embodied mismatch/prediction error and conditions consistent with reconsolidation-relevant updating, leading to altered stress-linked physiological prediction and potentially altered physiological and/or functional outcomes. The model is conceptually informed by predictive processing, interoceptive inference, allostatic predictive regulation, and reconsolidation-updating frameworks [6-9,17,18]. The figure is hypothesis-generating and should not be interpreted as evidence that NET induces prediction error, memory reconsolidation, or physiological updating. Credit: This figure was created manually by the author using Microsoft PowerPoint (Microsoft Corp., Redmond, WA) shapes and text only.

This interpretation is consistent with NET studies showing changes in emotional reactivity and brain physiology when distressing memories or emotionally salient stimuli are activated during treatment [2,20]. The proposed somatic-emotional updating sequence is illustrated in Figure 2.

The Somatic Imprint Model provides a useful conceptual bridge. This model describes how emotionally significant stressors may become embodied through somatic memory, stress imprinting, emotional conditioning, and trauma echoes [10]. Somatic-emotional updating extends this logic by proposing a possible mechanism through which such embodied patterns may become reactivated, destabilised, and modified during NET.

If NET updates emotionally encoded physiological predictions, then change should be observable not only in subjective distress but also in autonomic, behavioural, and biological markers over time. The proposed construct becomes clearer when mapped onto the sequence of reactivation, prediction error, lability, and updating described in reconsolidation research.

Mapping NET onto reconsolidation phases

The proposed somatic-emotional updating model can be clarified by mapping the NET clinical sequence onto the major phases described in memory reconsolidation theory. This mapping is intended as a hypothesis-generating framework rather than evidence that NET induces reconsolidation. It identifies points of conceptual overlap between NET, emotional memory reactivation, embodied mismatch, and subsequent updating and provides a framework for future empirical testing.

Reconsolidation research generally identifies three clinically relevant phases: reactivation of the target memory, mismatch or prediction error, and subsequent updating during restabilisation. In NET, these phases may correspond to activation of an emotionally salient stress pattern, introduction of contradictory embodied experience, and alteration of the associated psychophysiological response. This model is consistent with reconsolidation literature suggesting that retrieval alone may be insufficient for destabilisation and that prediction error may be required for an existing memory model to become modifiable [5-7].

During the reactivation phase, NET begins with a current concern, symptom, or stress-related presentation. Through the protocol, an associated emotional reality is identified and held in awareness. This may involve a related memory, phrase, image, bodily sensation, or affective state. From a reconsolidation perspective, the relevant target is not merely verbal recall but reactivation of a broader somatic-emotional network involving affect, interoception, posture, autonomic state, and conditioned threat response [8,21].

The second phase concerns prediction error, expressed clinically here as embodied mismatch. In reconsolidation research, prediction error refers to a mismatch between expected and actual experience. In NET, this mismatch is best understood as emerging from the full clinical context rather than from spinal or somatosensory stimulation alone. The patient contacts an emotionally charged reality while experiencing present-time safety, practitioner support, somatic attention, and non-threatening sensory input. The old prediction may involve overwhelm, shame, helplessness, danger, collapse, or dysregulation. The new experience is that the emotional reality can be contacted while the patient remains present, supported, and regulated [6,16].

If this mismatch is sufficient, the somatic-emotional pattern may become labile and open to updating. Within this model, updating may be reflected clinically by changes in emotional charge, breathing, posture, affect, autonomic tone, or subjective distress. These observations do not confirm reconsolidation, but they provide candidate markers for future research. The central question is whether such changes are durable and whether they correspond to measurable shifts in psychophysiological regulation.

The proposed NET-reconsolidation sequence can therefore be summarised as follows: a current stress response is identified, a related emotional reality is activated, the pattern is contacted within a safe clinical context, the expected emotional or physiological outcome is contradicted, and the stress-linked physiological prediction may reduce, reorganise, or lose threat value. The more precise hypothesis is that NET may create conditions consistent with the updating of emotionally encoded physiological predictions.

Table 1 summarises the proposed correspondence between reconsolidation phases and the NET clinical sequence.

**Table 1:** Proposed mapping of NET onto reconsolidation phases This table summarises hypothesised conceptual overlap between major reconsolidation phases and the proposed NET clinical sequence. Citations in the neuroscience description column refer to the underlying literature on reconsolidation and therapeutic change. The final column identifies empirical implications for future research. NET: Neuro-emotional technique. Credit: The author of this study, Ryan R. Day.

Reconsolidation phase	Neuroscience description	Proposed NET equivalent	Future empirical implication
Reactivation	Emotional memory network becomes active [5,7,21].	Emotional reality identified and held in awareness.	Durable change should require activation of the target somatic-emotional pattern, not general relaxation alone.
Prediction error/embodied mismatch	The expected emotional or physiological outcome of the reactivated memory is contradicted [6,16].	Emotional activation occurs with present-time safety, practitioner support, and somatosensory input.	A greater mismatch between expected threat response and actual safe experience should predict stronger clinical change.
Labile state	Memory may become temporarily modifiable [5-7,16].	Somatic-emotional pattern may destabilise.	In-session change should follow emotional activation plus mismatch, rather than recall alone.
Updating	Memory restabilises with new information [5,7,16,21].	Reduced threat value or altered physiological prediction.	Change should persist at follow-up rather than reflect only transient emotional relief

Table 1 is intended to define testable implications of the proposed NET-reconsolidation sequence rather than to catalogue all outcome domains examined in the existing NET research. Neurophysiological, endocrine, inflammatory, metabolic, pain, and functional changes reported in NET clinical studies and case reports are better interpreted as downstream candidate outcomes rather than direct evidence of reconsolidation phase transitions [2-4,20,22,23]. This distinction is important because biological or neurological change may be consistent with somatic-emotional updating without proving that reconsolidation has occurred.

A key question, therefore, is how the spinal or somatosensory component of NET might contribute to the proposed mismatch environment while the target somatic-emotional pattern is active.

Proposed role of spinal and somatosensory stimulation

A distinctive feature of NET is that emotional activation is paired with brief spinal or somatosensory stimulation. In practice, this may involve gentle manual tapping over specific spinal regions, instrument-assisted stimulation near the spinous processes, or related somatic input applied while the patient maintains awareness of the identified emotional reality. Within the present model, this component is not conceptualised as a mechanical release of emotion. Rather, it is proposed to provide novel, non-threatening sensory input while the target somatic-emotional pattern is active.

The timing of this input is central to the hypothesis. Somatosensory stimulation delivered outside emotional activation may function as a general manual or sensory intervention. In NET, however, the sensory input is paired with an emotionally salient pattern that has already been brought into awareness. The proposed relevance of spinal or somatosensory input, therefore, lies not only in the stimulus itself but in its delivery during a specific emotional and physiological state.

This interpretation is consistent with broader manual therapy literature suggesting that hands-on interventions may influence clinical outcomes through neurophysiological, contextual, and sensorimotor mechanisms rather than through biomechanical correction alone [24]. It is also supported by research indicating that spinal manipulation can alter central sensorimotor processing. Lelic et al. reported changes in prefrontal cortical processing after manipulation of dysfunctional spinal joints, suggesting that spinal sensory input may influence neural systems involved in sensorimotor integration and regulation [25].

NET-specific evidence also supports the potential relevance of the spinal stimulation component. Peterson compared NET delivered with spinal stimulation to a sham condition in which the same procedure was performed without spinal force, while phobic participants were exposed to a threat stimulus. Emotional arousal decreased significantly only in the spinal stimulation group, suggesting that somatosensory input delivered during emotional activation may contribute to reductions in threat reactivity [20]. This was a single controlled trial, and its findings require independent replication before firm conclusions can be drawn. Although this study does not establish a reconsolidation mechanism, it supports the hypothesis that spinal or somatosensory input may be clinically relevant when paired with an active emotional response.

From a predictive processing perspective, such input may contribute to embodied mismatch, proposed here as the clinical expression of prediction error during NET. The emotionally activated pattern may predict threat, overwhelm, helplessness, shame, collapse, or dysregulation, while the actual experience includes present-time safety, bodily awareness, and non-threatening sensory input. This contrast may signal that the present context differs from the original emotional learning. In this sense, spinal or somatosensory stimulation is not the whole prediction error, but one component of a wider mismatch environment [6,9].

The spinal or somatosensory component may therefore serve several related functions: increasing salience, introducing sensory novelty, supporting present-moment orientation, and providing interoceptive contrast during emotional activation. This framing allows the spinal stimulation component of NET to remain mechanistically meaningful as one contributor to a broader mismatch environment. The hypothesis can be tested by comparing full NET with modified protocols that preserve emotional activation but omit spinal or somatosensory stimulation or by comparing somatosensory input alone with the full clinical sequence.

If somatic-emotional updating alters the threat value of a stress-linked pattern, the effects may plausibly extend beyond emotional reactivity into broader domains of adaptive physiology.

Adaptive physiology and the psycho-immune-neuroendocrine (PINE) network

The proposed somatic-emotional updating model becomes more clinically meaningful when considered within the broader context of adaptive physiology. Emotional stress is not confined to subjective distress or a conscious mood state. It can influence autonomic regulation, neuroendocrine activity, immune signalling, inflammatory tone, pain sensitivity, sleep, behaviour, recovery capacity, and metabolic function. These interacting systems are commonly described through psychoneuroimmunology or PINE frameworks.

Within a PINE framework, psychological meaning, neural processing, endocrine signalling, and immune regulation are understood as interdependent elements of biological adaptation. Stress responses may be protective when they are brief, proportionate, and contextually appropriate. However, repeated or unresolved activation may contribute to allostatic load, impaired regulation, and physiological strain. McEwen and Seeman described this distinction by showing that stress mediators can support adaptation in the short term but may become damaging when repeatedly or chronically activated [1].

This is relevant to NET because the proposed target is not merely emotional distress but a stress-linked physiological prediction. If a somatic-emotional pattern continues to predict threat, helplessness, shame, loss, or danger, the body may continue to prepare defensively even when the original context is no longer present. This may be expressed through altered autonomic tone, heightened vigilance, disrupted sleep, pain amplification, inflammatory signalling, endocrine shifts, or maladaptive behavioural responses. The model does not assume that physiological strain is caused by emotion alone. Rather, it proposes that emotional learning may contribute to biological regulation through known stress pathways.

Existing NET research provides preliminary support for this biological framing. The randomised controlled trial by Bablis et al. reported improvements in chronic low back pain outcomes alongside changes in inflammatory and biobehavioural indicators after NET care [3]. Similarly, a type 2 diabetes and stress case report described changes in psychometric measures and blood chemistry following NET care, supporting the rationale for studying NET within stress physiology and PINE frameworks [22]. These findings do not prove memory reconsolidation. They do, however, suggest that NET effects may extend beyond subjective emotional change into measurable biological regulation.

Neuroimaging studies also support the relevance of this model. Monti et al. reported reduced activation in regions involved in traumatic memory and distress, including the parahippocampus, brainstem, anterior cingulate, and insula, after NET in cancer survivors with traumatic stress symptoms. A related study found changes in cerebellar functional connectivity with limbic and brainstem regions, alongside reduced autonomic reactivity to a traumatic stimulus [2,23]. These findings also do not prove reconsolidation, but they support the plausibility that NET may influence emotional memory networks and autonomic regulation.

Psychoneuroimmunology further supports this direction. Chronic psychological stress can alter inflammatory regulation, and immune function is sensitive to social, emotional, and contextual signals. Social Safety Theory proposes that cues of threat and safety are biologically meaningful and may influence inflammatory activity and health risk [26]. This supports the plausibility that emotionally encoded threat predictions may influence adaptive physiology through autonomic, neuroendocrine, and immune pathways.

The implication is not that NET directly treats inflammatory, endocrine, metabolic, or immune disease. A more precise hypothesis is that some stress-linked physiological patterns may be maintained by maladaptive somatic-emotional predictions and that updating those predictions may reduce repeated defensive physiological activation. If this model is correct, future studies should be able to detect NET-associated changes not only through changes in subjective distress but also through durable shifts in autonomic regulation, biological stress markers, sleep, behaviour, recovery, and functional capacity.

Table 2 outlines the proposed pathway from somatic-emotional updating to measurable domains of adaptive physiology.

**Table 2:** Candidate outcome domains for future studies of somatic-emotional updating This table presents candidate outcome domains for future studies of somatic-emotional updating. Row-level citations identify literature supporting the relevant biological domain, prior NET findings, or candidate measurement areas. The table represents a conceptual hypothesis and should not be interpreted as evidence that NET has been shown to produce changes across all listed measures. CRP: C-reactive protein; DHEA-S: Dehydroepiandrosterone sulphate; HRV: Heart rate variability; IL: Interleukin; NET: Neuro-emotional technique; TNF: Tumour necrosis factor. Credit: The author of this study, Ryan R. Day.

Level	Proposed process	Candidate outcome measures for future research
Emotional	Reduced threat appraisal linked to target pattern [21,26]	Target distress, anxiety, emotional reactivity, and subjective emotional charge [2,20]
Autonomic	Altered autonomic tone and stress reactivity [1,18,23]	HRV, heart rate, skin conductance, and respiratory rate [23]
Neuroendocrine	Altered stress-axis signalling [1,18]	Diurnal cortisol, cortisol-awakening response, DHEA-S, and cortisol:DHEA ratio [1]
Immune/inflammatory	Modulation of stress-linked inflammatory signalling [1,3,26]	CRP, TNF-alpha, IL-1, IL-6, IL-10, and other cytokines [3,26]
Behavioural	Improved stress-related behavioural regulation [26]	Sleep, activity, avoidance behaviour, and adherence [26]
Functional	Improved adaptive capacity under load [1,3]	Pain and disability [3], quality of life, recovery capacity, and functional capacity

This adaptive physiology framing also helps position NET alongside other interventions that combine emotional activation, sensory experience, and corrective learning.

Distinguishing NET from related therapies

The proposed somatic-emotional updating model places NET within a broader group of interventions that engage emotional memory, bodily awareness, corrective experience, and physiological regulation. However, NET should not be understood as identical to psychotherapy, exposure therapy, eye movement desensitization and reprocessing (EMDR), or somatic trauma therapy. It is better described as a distinct mind-body protocol that integrates emotional activation, interoceptive attention, psychophysiological cueing, and spinal or somatosensory input within a structured clinical sequence.

Several psychotherapeutic models overlap conceptually with memory reconsolidation theory. Lane et al. proposed that therapeutic change across multiple modalities may involve emotional arousal, activation of prior emotional memories, and incorporation of new emotional experience through reconsolidation. This provides a useful parallel for NET because it suggests that durable change may require more than insight, relaxation, or symptom suppression. It may require reactivation of old emotional learning in the presence of new experiential information [21].

EMDR provides a relevant comparison because it pairs distressing memory material with structured sensory input. This does not imply that NET and EMDR necessarily share the same mechanism. Rather, it shows that sensory input can form part of a recognised memory-processing intervention. NET differs from EMDR because it emerged from chiropractic and applied kinesiology traditions and uses manual muscle testing, emotional reality identification, and spinal or somatosensory stimulation as part of its protocol [27].

Exposure therapy provides a second useful comparison because modern exposure models often explain clinical improvement through inhibitory safety learning rather than direct modification of the original fear memory [28]. This provides an important boundary condition for NET and is considered further in the following section.

Somatic therapies provide a further comparison because they emphasise interoception, proprioception, autonomic state, and the bodily expression of stress. NET shares this broader somatic orientation but differs through its standardised sequence, use of psychophysiological cueing, and brief spinal or somatosensory input during emotional activation. Across these comparisons, NET appears best understood as a distinct mind-body protocol whose proposed relevance lies in the structured combination of emotional activation, bodily attention, contextual safety, and somatosensory input.

These comparisons clarify NET’s distinctive structure and provide a rationale for considering competing explanations before defining the model’s limitations.

Competing explanatory models

Somatic-emotional updating is proposed in this study as a hypothesis-generating model, not as the only plausible explanation for clinical changes reported following NET. Several alternative or complementary mechanisms may account for some or all of the reported effects.

First, change may occur through extinction learning or inhibitory learning. In this account, activation of emotional material in a safe context may create new safety learning that competes with the original response rather than modifying the original emotional learning itself [28]. Second, cognitive reappraisal may contribute when the patient develops a new interpretation of the emotional theme, memory, body sensation, or personal meaning associated with the presenting concern [29]. Third, expectancy effects and placebo-related mechanisms may influence symptoms, affect, pain, autonomic state, or perceived improvement through treatment context, prior belief, practitioner confidence, and patient expectation [30]. Fourth, therapeutic alliance and contextual safety may contribute through the effects of being supported, attended to, and guided by a clinician within a structured therapeutic encounter [31].

These explanations are not mutually exclusive. NET may involve multiple processes, including emotional activation, attentional redirection, autonomic settling, somatosensory input, cognitive meaning-making, expectancy effects, and relational safety. The reconsolidation-relevant model proposed here would be strengthened if future studies can show durable, target-specific changes that depend on emotional activation plus embodied mismatch and that are not equally explained by supportive interaction, recall alone, general relaxation, or expectancy effects.

Limitations and boundary conditions

The proposed somatic-emotional updating model requires several empirical boundary conditions. Although NET contains elements that appear compatible with memory reconsolidation, including emotional activation, embodied mismatch, somatosensory input, and reassessment, no study has directly tested whether NET induces memory destabilisation or reconsolidation.

A first limitation concerns the translation of reconsolidation research into complex clinical practice. Much of the strongest reconsolidation evidence comes from controlled experimental paradigms, including fear-conditioning and laboratory memory-updating studies. Clinical emotional memories are more complex and may include autobiographical memory, bodily state, relational meaning, interoceptive prediction, conditioned autonomic response, and narrative interpretation [7,16].

A second limitation concerns prediction error. Reconsolidation research suggests that retrieval alone is often insufficient, but prediction error remains difficult to define and measure clinically. In NET, the proposed mismatch involves emotional activation, present-time safety, practitioner support, interoceptive attention, and somatosensory input. Future studies will need to define measurable indicators of embodied mismatch rather than assuming that prediction error occurs whenever the protocol is applied.

A third limitation concerns mechanism specificity. As discussed above, reconsolidation is only one possible explanation for clinical change following NET. Future studies should test whether observed changes are durable, target-specific, and dependent on emotional activation plus embodied mismatch rather than assuming that improvement necessarily reflects reconsolidation-relevant updating. The model must also avoid implying that recalled emotional material is historically accurate. The target of the proposed mechanism is the current somatic-emotional prediction, not the factual validation of a recalled event [32].

A fourth limitation concerns the clinical literature base for NET specifically. Published NET research includes randomised and controlled clinical studies, neuroimaging studies, pilot studies, and case reports across a range of conditions. While this body of work demonstrates meaningful breadth, it remains modest in total volume and has been produced predominantly by a small number of research groups with professional or clinical investment in the technique. Independent replication by researchers without prior commitment to NET would substantially strengthen the evidence base and reduce the risk of allegiance bias.

Finally, biological and neurological changes observed in NET studies do not by themselves prove reconsolidation. Physiological changes may occur through multiple pathways. Somatic-emotional updating is proposed as one possible mechanism that may apply to selected patients and selected stress-linked patterns. Its clinical value will depend on whether future studies can identify when, for whom, and under what conditions NET produces durable changes in emotional reactivity, autonomic regulation, and adaptive physiology.

Future research directions

The somatic-emotional updating model generates several testable predictions. If reconsolidation-relevant processes contribute to NET outcomes, emotional reactivation should be necessary, embodied mismatch should predict outcome strength, and clinical changes should persist beyond immediate post-session relief. These predictions allow the model to be tested empirically rather than treated as a purely theoretical explanation.

Future mechanism-focused studies could build on existing NET research by integrating measures that have previously been studied separately, including target emotional distress, symptom-specific outcomes, autonomic markers, inflammatory markers, endocrine or metabolic markers, sleep measures, and quality-of-life scores. These measures could be collected before NET, immediately after NET, and at follow-up intervals such as one week, one month, and three months. This would help determine whether NET-associated changes are durable, target-specific, and measurable across subjective, autonomic, biological, and functional domains.

In addition to outcome measurement, future research should attempt to operationalise prediction error. This could include measuring the patient’s expected emotional or physiological response before reactivation and comparing it with the actual experience during the NET process. Comparative studies should test the full NET against modified procedures that isolate or omit key elements of the proposed mismatch environment. Such designs would help distinguish reconsolidation-relevant updating from extinction learning, expectancy effects, contextual safety, general relaxation, or non-specific therapeutic interaction.

A further research priority is a dedicated scoping review of the NET evidence base. Such a review could map published NET studies by study design, population, clinical indication, intervention characteristics, outcome domains, quantitative findings, and methodological limitations. Where sufficient homogeneity exists, later systematic reviews could evaluate efficacy or specific mechanistic hypotheses.

## Conclusions

NET is a structured mind-body intervention whose mechanisms remain incompletely defined. This review proposes somatic-emotional updating as a hypothesis to explain how emotional activation, psychophysiological cueing, contextual safety, interoceptive attention, and non-threatening somatosensory input may contribute to embodied mismatch and to changes in stress-linked physiological prediction patterns. Memory reconsolidation and predictive processing provide the theoretical basis for this model. Still, the model should not be interpreted as evidence that NET induces prediction error, memory reconsolidation, or physiological updating. Future studies should test whether NET-associated changes are durable, target-specific, physiologically measurable, and dependent on the proposed mismatch conditions and whether they are reflected in emotional reactivity, autonomic regulation, biological stress markers, and functional outcomes.
